# The Medical Schools of Baltimore

**Published:** 1880-11

**Authors:** Eugene F. Cordell

**Affiliations:** Baltimore, Md.


ARTICLE III.

### THE MEDICAL SCHOOLS OF BALTIMORE.

BY EUGENE F. CORDELL, M. D., BALTIMORE, MD.

[We are under obligation to the author for his politeness in kindly allowing the following interesting abstract of his valuable paper, read by appointment before the Medico-Chirurgical Society, at its recent meeting in this city, to appear in the present issue of the journal.]

Dr. Cordell read the next paper on the "Medical Schools of Baltimore," of which we offer to our readers the following abstract: He began by a reference to the importance of *medical education.* "It lies at the root of the whole system of human activity, of which we are the representatives, and the character of the super-structure is dependent in no small degree upon the foundation upon which it rests. The attachment to the institutions in which we have received our training, gives the subject a more personal interest, although it is true in a more restricted field. Its interest is enhanced at this time by the reform movement which has recently set in in this country, and is making sure progress. He regretted that he could not yet record the workings of this movement in Baltimore. He could not sympathise, however, with those pedants, who, having adopted measures of reform, look with contempt or indifference upon the entire past, which contains so much that is excellent, so much that is great in sentiment and achievement.

In considering medical education as it has existed in Baltimore during the century and a half of its history, brief allusion was made to unsuccessful attempts to establish medical schools in 1789, 1797 and 1803. Also to a "Collegicum Medicorum," which according to a diploma in the possession of Dr. John Morris of this city, was instituted in 1801, but of which a careful investigation had failed to elicit any satisfactory information.

The first medical school in Baltimore had its origin in an Act of the Legislature of Maryland passed Dec. 18th, 1801, and owed its existence chiefly to the labors and influence of Dr. John Beale Davidge, who had been giving private courses of lectures for some years previously. D. was ably assisted by Drs. James Cocke of Virginia, and John Shaw, of Annapolis. The name of this institution was the "College of Medicine of Maryland."

The difficulties encountered by its founders were at first almost insurmountable, but by the zeal and liberality of the faculty and the help of a lottery for which authority was obtained from the state, the imposing and substantial building on the corner of Lombard and Green streets, was erected in 1812-13, at which time also, the institution was converted into a University with Faculties of Law, Theology, Arts and Sciences, the whole constituting a Board of Regents, into whose hands the affairs of the institution, as re-organized, were entrusted.

Success was now assured, and the class increased in numbers yearly until in 1825-27 it numbered over 300; meanwhile, "Practice Hall" and the "Baltimore Infirmary" had been created, and a museum established, by the purchase of the valuable pathological collection of Prof. Allen Burns of Glasgow.

At this time, (1822,) the school had, probably, no superior on the continent. But the Legislature now saw fit to abolish the Board of Regents, and to transfer the authority to a Board of Trustees, composed of non-medical men entirely. This act, (which violated the sacred rights of private ownership and was totally unjustifiable,) was resisted by the Medical Faculty; but in vain, until 1837, when the long half-smothered and but ill-concealed sense of injury and injustice burst forth into open opposition, and suit was instituted by the surviving regents for the recovery of their property and rights. After two years of litigation, the Court of Appeals decided in their favor, and the entire control of the institution was restored to their hands. The school had lost ground greatly in this period, and the class had been reduced to 18 matriculants in 1839.

On the restoration of their rights the faculty made renewed efforts, the school began once more to flourish, and except during the period of the war, (when the southern patronage was cut off,) has slowly but steadily improved to the present time, when it ranks as one of the leading metropolitan schools of America. Among the most distinguished members of its faculties, have been Nathan R. Smith, Nathaniel Potter, John B. Davidge, Elisha Bartlett, Elisha DeButts, William Gibson, Granville Sharp Pattison, Eli Geddings, Robley Dunglison, Robt. E. Griffith, Joseph Roby, William A. Hammond and Charles Frick.

Among its distinguished alumni have been Horatio G. Jameson, Lunsford P. Yandell, John D. Godman, James B. Rogers, Wm. Power, M. M. Pallen, John H. Harrison, James L. Cabell, Louis A. Dugas, Augustus L. Warner, Thomas E. Bond, Thomas H. Buckler, Samuel Chew, Charles Frick, Roberts Bartholow, Nathan S. Lincoln, Surgeon-General Wales, &c. The whole number of alumni amounts to about 3,200. The number of students in attendance upon the course of 1879-80, was 173, of whom 66 received the degree of M. D.*

*The author of the paper is engaged in preparing an elaborate "Historical Sketch of Medicine of the University of Maryland," which will be published sometime during the coming winter.

The next school instituted in Baltimore was the "Washington Medical College, which was founded in 1827, chiefly through the exertions of Dr. Horatio G. Jameson, the eminent surgeon. The authority to confer degrees was derived from Washington College, Washington, Pennsylvania." Under the zealous management of a faculty, which included some very able names, the institution soon began to acquire prominence and to enter into competition with older schools. The building erected for its special use, on Holliday Street, opposite the old City Hall, soon proved insufficient for the needs of the growing classes, and another was erected on Broadway (the present "Church Home and Infirmary.")

About 1838, the College was converted by act of the Legislature, into an University with the usual additional Faculties. This did not make any material changes, however, as the other departments never had any other than a mere nominal existence.

Experience demonstrated that the situation on Broadway was too remote from the centre of the City's population and the Faculty with a zeal and enterprise that seems almost incredible, determined to erect a third building in a more accessible locality, and to this end contributed from their private funds liberally. The corner of Hanover and Lombard Streets was selected and the "New Assembly Rooms" ( since used by the Baltimore Dental College, and the College of Physicians and Surgeons,) was built. But either from the magnitude of enterprise or from mismanagement of the funds on the part of those to whom they were entrusted ( as was alleged, ) the undertaking over-taxed the resources of the Faculty to such a degree, that the building had to be sold immediately, in order to enable them to meet the obligation incured in its erection. This occured in 1851, and cut short the career of the school.

From this time until 1866, it remained in an inactive condition, no effort being made to re-establish it. In 1866, it was revived, chiefly owing to the efforts of Dr. Harvey L. Byrd, Dr. Thomas E. Bond, and Dr. Edward Warren,

The surviving members of the old Faculty still held on to the charter, and as the representatives of the Medical Department, had power to transfer their official rights to such persons as they chose to elect as their successors. In this way a new Faculty was created, and the school again brought to life. The lectures were at first delivered in the large building on the N. E. corner of Calvert and Saratoga Streets, but after one or two sessions the building on the opposite corner was secured, and by the aid of an appropriation from the state, converted into a hospital, with lecture rooms, &c.

For some years the prospects of the Institution were encourageing, and met with considerable success, but in 1872, some differences arose in the Faculty, which led to the resignation of Dr. Warren, who joined with some others in the formation of a third medical school—the "College of Physicians and Surgeons," in the charter of which Drs. Warren, Byrd, Opie, Lynch, Murray and Goolrich are named as the first Faculty.

The Washington University now began evidently to lose ground, whilst the new College exhibited constantly increasing vitality and success. In the Spring of 1877 the two were merged in conformity with an act of Legislature, under the name of the "College of Physicians and Surgeons." The hospital, on the corner of Calvert and Saratoga Streets, (since known as the City Hospital,) became the property of the consolidated Institution.

The total number of graduates of the Washington University, during the stages of its existence, was about 700.

The conclusion, derived from a general survey of the field passed in the paper, was that the Medical Schools of Baltimore in the past, would compare favorably with those of most other cities in America. A hope was expressed that the historian of the Bi-Centennial Celebration would be able to utter the same opinion with regard to the coming time.

